# Intracytoplasmic Re-localization of miRISC Complexes

**DOI:** 10.3389/fgene.2018.00403

**Published:** 2018-09-20

**Authors:** Bünyamin Akgül, İpek Erdoğan

**Affiliations:** Non-coding RNA Laboratory, Department of Molecular Biology and Genetics, İzmir Institute of Technology, Urla, Turkey

**Keywords:** microRNA, localization, polysome, mRNP, translational repression

## Abstract

MicroRNAs (miRNAs) are a conserved class of non-coding RNAs of 22 nucleotides that post-transcriptionally regulate gene expression through translational repression and/or mRNA degradation. A great progress has been made regarding miRNA biogenesis and miRNA-mediated gene regulation. Additionally, an ample amount of information exists with respect to the regulation of miRNAs. However, the cytoplasmic localization of miRNAs and its effect on gene regulatory output is still in progress. We provide a current review of the cytoplasmic miRNA localization in metazoans. We then discuss the dynamic changes in the intracytoplasmic localization of miRNAs as a means to regulate their silencing activity. We then conclude our discussion with the potential molecules that could modulate miRNA localization.

## Introduction

MicroRNAs (miRNAs) are an important group of non-coding RNAs (ncRNAs) of 17–22 nt in length that regulate gene expression post-transcriptionally typically by binding to the 3′ untranslated regions (UTR) of mRNAs (Bartel, 2018). The first example of miRNAs, lin-4, was identified in *C. elegans* during a screen for genes that are important for developmental timing (Lee et al., 1993; Wightman et al., 1993). Expressed in a variety of species ranging from viruses to primates (Griffiths-Jones et al., 2006), miRNAs are now recognized as micromanagers of post-transcriptional gene expression both in development and diseases (Bartel and Chen, 2004).

**FIGURE 1:**
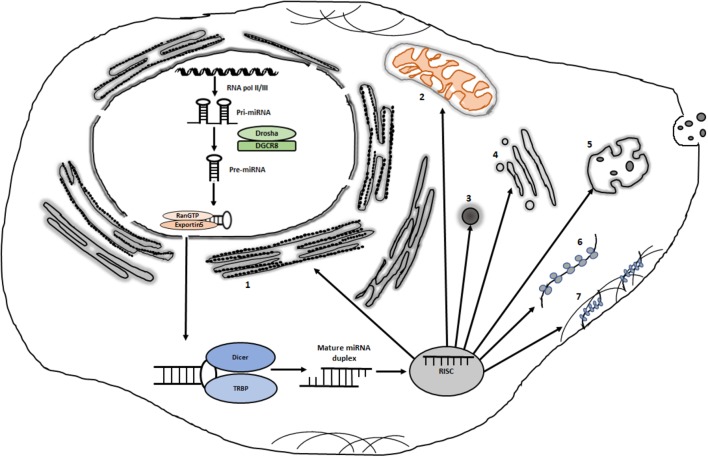
Potential intracytoplasmic destinations of miRISCs. Following transcription and processing in the nucleus, some miRISCs are destined for the nucleus (not shown). Cytoplasmically localized miRISCs can be localized in distinct sites such as: (1) rough endoplasmic reticulum; (2) mitochondria; (3) various types of messenger ribonucleoprotein granules such as P bodies and stress granules; (4) golgi; (5) multivesicular bodies; (6) free polysomes and (7) cytoskeleton-bound polysomes. Organelles and complexes are not drawn to scale.

In the canonical pathway (**Figure 1**), miRNA genes are transcribed by RNA polymerase II with a 5′ cap and a 3′ tail (Lee et al., 2004). The first processing step takes place in the nucleus where the primary miRNA transcript is trimmed into a stem-loop structure by Drosha (Lee et al., 2003). The stem-loop structure with a 2 nt overhang is then transported to the cytoplasm through Exportin-5 (Lund et al., 2004) where it is converted into a 22 nt duplex by Dicer (Chendrimada et al., 2005). The strand with the more unstable 5′-end is usually selected for loading onto miRNA-induced silencing complex (miRISC) (Iwasaki et al., 2010). miRISC is a microribonucleoprotein complex that are composed of AGO proteins, glycine-tryptophan protein of 182 kDa (GW182) and some other proteins (Krol et al., 2010). Based on the complementarity between the miRNA and its target, RISC-bound miRNAs induce target RNA degradation and/or translational suppression (Yekta et al., 2004; Filipowicz et al., 2008). The carboxy-terminal part of GW182 is involved in the recruitment of poly A binding protein (PABP) and the deadenylases CCR4 and CAF1. mRNA degradation is initiated through deadenylation by the PAN2-PAN3 and CCR4-NOT complexes followed by DCP2-mediated decapping and degradation by the 5′–3′ exonuclease XRN1 (Jonas and Izaurralde, 2015). In this process, GW182/TNRC6 proteins coordinate interactions between AGOs and the PAN2-PAN3 and CCR4-NOT complexes. The CCR4-NOT complex may also induce translational repression but the repression may also involve the release of eIF4A1 and eIF4A2 from target mRNAs (Fukao et al., 2014; Fukaya et al., 2014). miRISCs contain a number of additional factors that might modulate miRNA activity or miRISC localization (Zhang et al., 2007; Krol et al., 2010). miRNAs may also be generated through alternative pathways, such as Drosha-DGRC8-independent and TUTase-dependent pathways (Yang and Lai, 2011). In this review, we will discuss miRNA localization, focusing on polysomal association and re-distribution of polysome-associated miRNAs as a means to regulate miRNA silencing activity.

## miRNA Localization

miRNAs, miRNA-targeted mRNAs and associated proteins were traced to determine the intracellular location of miRISCs. Earlier studies that involved injection of labeled miRNAs or *in situ* hybridization revealed that miRISC is primarily localized in the cytoplasm (Pillai et al., 2005; Bhattacharyya et al., 2006). The use of luciferase reporter mRNA with miRNA binding sites showed localization of target mRNA to processing bodies (PBs) in a miRNA-dependent way (Liu et al., 2005; Pillai et al., 2005). These studies also detected AGO proteins in the cytoplasm, clearly documenting the cytoplasmic localization of tested miRNAs, their targets and associated proteins. Considering the fact that miRNA maturation and loading onto AGO proteins occur in the cytoplasm, the cytoplasmic localization of miRISC has been widely accepted as a general rule of thumb. P bodies, stress granules (SGs), multivesicular bodies (MVBs), endoplasmic reticulum (ER) and mitochondria are considered to house miRISCs in the cytosol (Leung, 2015). Interestingly, genome-wide small RNA profiling studies demonstrated that mature miRNAs exist in the nucleus and associate with AGO proteins (Meister et al., 2004; Jian-You et al., 2010; Jeffries et al., 2011). We will primarily focus on the cytoplasmic re-arrangement of miRISC and thus refer the readers to the excellent reviews that deal with nuclear functions of miRNAs (Catalanotto et al., 2016; Liu et al., 2018).

## Non-Membraneous Cytoplasmic Foci

Cytoplasm contains various non-membraneous compartments that may serve as important sites for RNA biology, including miRNA-mediated gene regulation. Among the best characterized non-membraneous compartments are PBs, SGs, germ granules, and neuronal granules (**Figure 1**).

Processing bodies, which contain various proteins associated with mRNA decay, translational control and RNA interference, were termed based on their content of proteins to carry out decapping of mRNAs (Sheth and Parker, 2003). Both AGO and GW182 proteins co-localize with PBs (Liu et al., 2005). In earlier studies on EGFP-AGO2-transfected cells under non-physiological forced expression conditions, approximately 1.3% of the total fluorescence intensity stemmed from PBs (Leung et al., 2006), suggesting the existence of other sites for miRNA-mediated translational repression or mRNA decay (Leung and Sharp, 2013). Although these data point to the possibility that PBs are probably not the sole sites of action for miRNA-mediated gene regulation, a plethora of evidence exists for PBs as storage sites of miRNA targets (Bhattacharyya et al., 2006; Krol et al., 2010; Jonas and Izaurralde, 2015). Although the absence of poly(A) tails in PB-stored mRNAs questions the involvement of PBs in miRNA-mediated storage of target mRNAs and their subsequent translation (Aizer et al., 2014), a fluorescence-activated particle sorting of endogenous PBs provided strong evidence for GW182-dependent, AGO2-mediated translational repression in P bodies (Hubstenberger et al., 2017).

Stress granules are messenger ribonunleoprotein (mRNP) granules that include mRNAs stalled in translation initiation or disassembling from polysomes (Protter and Parker, 2016). Thus, stress granules are characterized by the presence of translation initiation factors and RNA-binding proteins as well as 40S ribosomal subunits (Anderson and Kedersha, 2008). Stress granules are formed mainly under stress conditions as an adaptive response by temporary and global stall in translation of cellular mRNAs with the aim of optimizing RNA metabolism toward damage repair (Ron and Walter, 2007). One can then hypothesize that miRNA-mediated translationally blocked mRNAs could potentially end up in SGs until the stress invoking condition goes away. In fact, two important components of SGs, fragile X mental retardation protein and PABP1, interact with the miRNA machinery (Jin et al., 2004; Moretti et al., 2012). Along this line, quantitative analysis of the kinetics of AGO2 localization revealed that AGO2 is normally distributed diffusely in the cytoplasm under physiological conditions but is recurited into newly formed SGs under stress conditions (Leung et al., 2006). However, more studies are required to delineate the fate of AGO-interacting mRNAs in the SGs.

Germ granules are another example of mRNP granules that are primarily associated with germ cell lineage functions (Gallo et al., 2008). miRISC components are known to co-purify with germ granule proteins (Wu et al., 2017). Interestingly, mRNP formation in germ granules involves scanning and recognition of target sites by miRISC followed by CCR4-NOT recruitment and particle nucleation. Neuronal granules are also a type of RNP granules that are formed in neurons as transport granules since neurons require long-distance transport of mRNAs along axons and dentrites (Kiebler and Bassell, 2006). The fact that translation of mRNAs is suppressed during the transport of mRNAs to their destination (Krichevsky and Kosik, 2001) raises the possibility that these mRNAs could be translationally stalled by miRISCs. Fragile X mental retardation protein is a key component of neuronal granules (Kiebler and Bassell, 2006) and its interaction with miRNAs and RISC suggests the localization of at least some miRNAs in neuronal granules (Zalfa et al., 2006).

## Endomembrane and Mitochondria for miRNA Action

Because SGs, germ granules, and neuronal granules are formed under special conditions or in special cell types and P bodies are not the sole sites of miRISCs in the cytoplasm, there must be other cytoplasmic foci that harbor miRISC complexes. Such potential foci are, but not limited to, endomembranes that include ER, golgi, and MVBs in addition to a non-membraneous organelle, mitochondria (Kim et al., 2014).

As an organelle specialized in protein translation, ribosomes on rough ER translates ER-bound proteins, which are docked onto the rough ER through the recognition of N-terminal signal peptides during active translation by the signal recognition particle (SRP) localized on the ER (Schwartz, 2007). ER could house at least the miRNAs that regulate gene expression at the protein translation level. In fact, serum starvation triggers the assembly of a number of miRISCs on polysome-bound mRNAs in Drosophila S2 cells and some of these miRISCs sediment together with the ER component (Wu et al., 2013). Also, the *AMP1* gene in *Arabidopsis* modulates miRNA-mediated gene expression and it is localized on the rough ER together with AGO1 (Li et al., 2013). A recent work by Sahoo et al., showed that annulate-lamellae-associated Nup358, which is a nucleoprotein, binds to AGO proteins and facilitates mRNA:miRISC intereaction through its SUMO-interacting motifs (Sahoo et al., 2017). Although Nup358 depletion has no detectable effect on AGO2 localization to rough ER, Nup358-positive annulate-lamellae structures are associated with PBs and SGs, indicating a potential role for annulate lamella in coordinating the cytoplasmic face of miRISCs. We would like to refer the readers to an excellent review by Kim et al. (2014), on connections between miRNA-mediated silencing and endomembranes that include ER, endosomes, MVBs, lysosomes, autophagosomes, vacuoles and golgi. Multivesicular bodies are the last stage of endosomes involved in the trafficking of molecules to lysosomes, plasma membrane or golgi for various purposes. Interference with the assembly or turnover of MVBs affects miRNA-mediated regulation, suggesting a potential role for MVBs in miRISC dynamics (Gibbings et al., 2009). Considering the importance of exosome-bound miRNAs in regulating gene expression in the recipient cells (Janas et al., 2015), MVBs could play a central role in the assembly and sorting of exosome-bound miRNAs. It remains to be unraveled how miRNAs are specifically targeted into late endosomes and more importantly what determines the cytoplasmic fate of late endosomes that contain different miRNA cargos.

An interesting site for the nuclear-encoded miRNAs is mitochondria (Kren et al., 2009). Both pre-miRNAs and mature miRNAs exist in mitochondria, suggesting the mitochondrial existence of miRNA biology (Barrey et al., 2011). However, only a fraction of miRNAs exist in mitochondria and they mainly coordinate mitochondria-related functions such as mitochondria morphology, mitochondrial metabolism and cell death (Li et al., 2012).

## Polysomal and Non-Polysomal Sites for mirna Action

The cellular translation machinery may be fractionated into different types of sub-fractions based on their translational status by taking advantage of sucrose-density fractionation (Tu and Akgül, 2005). In this approach, the cytosolic complexes are fractionated based on their molecular weight through centrifugation on a sucrose density gradient. Such a fractionation typically yields four sub-fractions, which are (1) translationally silent mRNP; (2) 40S/60S ribosomal subunits; (3) 80S monosomes, and (4) polysomes.

Polysomes are formed during active translation through the sequential assembly of ribosomes on mRNAs. Non-polysomal mRNA complexes typically carry less than two ribosomes while polysomes carry two or more ribosomes. Considered as the cellular translation machinery, polysomes are classified into three groups based on their intracytoplasmic location (1) free polysomes; (2) ER-bound polysomes, and (3) cytoskeleton-bound polysomes (Reid and Nicchitta, 2015). Accordingly, each type of polysome synthesizes different types of mRNAs. For example, free polysomes are involved in the translation of cytosolic proteins whereas cytoskeleton-bound polysomes primarily translate asymmetrically distributed mRNAs that are enriched in a subcellular location (Lerner et al., 2003).

Taking into account the fact that miRNA-mediated regulation partially involves translational repression and translation-coupled mRNA decay, the polysome-bound state of a miRNA could provide valuable information about its potential regulatory function. More importantly, recycling miRISCs between polysome-bound and non-polysome-bound (e.g., messenger ribonucleoprotein) target mRNA could be a potential regulatory mechanism. Although it has been challenging to uncouple mRNA degradation from translational repression, ribosome profiling has been used to demonstrate mRNA degradation to have the dominant effect in post-embryonic cells (Guo et al., 2010; Eichhorn et al., 2014). It is also widely accepted that miRNAs suppress translation at early points following their expression (Filipowicz et al., 2008). In fact, when a miRNA is over-expressed, translational repression precedes mRNA degradation (Béthune et al., 2012). However, by the time the full miRNA-mediated repression is manifested, mRNA destabilization takes over the silencing effect. Quantitative studies in mammalian cells showed that 6–26% of the repression occurs through translational repression without any contribution from mRNA degradation (Eichhorn et al., 2014). However, counting in the translation-coupled mRNA degradation, this percantage could be higher. On the other hand, miRNA-mediated silencing involves translation repression in embryos (Bazzini et al., 2012), suggesting that the consequence of miRNA repression is different in embryos probably due to the context of post-transcriptional regulatory mechanisms unique to embryos.

Polysome association of miRNAs was reported as early as 1999 (Olsen and Ambros, 1999). Later polysomal association of miRNAs has been reported in various mammalian cell types (Kim et al., 2004; Maroney et al., 2006; Nottrott et al., 2006; Petersen et al., 2006). However, not all miRNAs sediment with polysomes (Nelson et al., 2004), suggesting the presence of miRNAs on non-polysomal mRNPs. Disassembly of polysomes as a result of translational repression triggers the formation of mRNP complexes that sequester mRNAs in a translationally inactive state. P bodies and SGs (and probably neuronal granules and germ granules) are examples of such mRNPs that serve as a hub for sorting of mRNAs for their subsequent fates. It is widely accepted now that there is a functional link among polysomes, SGs and PBs (Protter and Parker, 2016; Chantarachot and Bailey-Serres, 2018). Re-arrangement, then, of the miRISC localization between different types of polyribosomes and mRNPs could be a regulatory mechanism to control miRISC activity. In fact, a switch between polysomes and exosomes has been proposed as a regulatory process in which miRISC localization is used to modulate miRNA silencing capacity. In this example, polysome association of miRNAs results in an impaired miRNA export, suggesting a potential regulatory mechanism that might involve re-arrangement of miRNA localization between exosomes and polysomes (Ghosh et al., 2015).

The long-standing dispute over miRNA-mediated translational repression at the translation initiation versus elongation has been resolved in favor of translation initiation (Pillai et al., 2005; Chu and Rana, 2006; Petersen et al., 2006; Jonas and Izaurralde, 2015). eIF4A1 and eIF4A2 are replaced by miRISC complex to inhibit translation initiation (Fukao et al., 2014; Fukaya et al., 2014). A major question related to miRNA-mediated translational repression is the site at which translational repression takes place although polysome-association is typically correlated with translational repression (Maroney et al., 2006). miRISCs appear to exist in high molecular weight complexes (HMWCs, e.g., polyribosomes) as well as low-molecular weight complexes (LMWCs, e.g., non-polysomal mRNPs) (La Rocca et al., 2015). Apparently, miRISC complexes exist in LMWCs in most healthy tissues while they exist in HMWCs in cell lines. Interestingly, in resting T cells, miRISCs are not actively engaged in target repression as they exist in LMWCs. Upon T cell activation, miRISCs are geared toward HMWCs (La Rocca et al., 2015). Messenger ribonucleoprotein complexes are dynamic structures that are formed from non-translating mRNAs through the binding of a number of proteins that determine the fate of mRNAs, e.g., translation, localization or turnover (Buchan, 2014). mRNPs are not merely formed as a consequence of polysome dissociation. Rather, the assembly of a fraction of it involves an orderly scanning and target site recognition by miRISC followed by the recruitment of additional factors such as the CCR4-NOT complex (Wu et al., 2017). Thus, as certain mRNP granules are formed as a result of polysome disassembly (e.g., under stress conditions), some could be formed *de novo* from non-translating mRNAs. Dynamics changes in the polysomal versus non-polysomal association of miRISCs suggests that the action site for each miRISC could be different based on its target, cell type or phenotypic context.

An in-dept analysis of small RNA profiling, preferably in conjuction with proteomics, based on their association with sucrose density fractions and comparison of such profiles between different conditions (e.g., different developmental stages or health versus disease) may provide insight into the potential role of polysomes in coordinating miRISC silencing activity. Göktaş et al. (2017) took advantage of the existence of extensive post-transcriptional gene regulatory networks in *Drosophila melanogaster* embryos to track the intracellular small RNA dynamics. Sucrose density fractionation of polysomes followed by small RNA profiling revealed (1) the presence of certain miRNAs non-selectively throughout all fractions; (2) the abundance of certain miRNAs in either polysomal or non-polysomal fractions; (3) the transition of certain miRNAs from polysomal fractions to non-polysomal ones or vice versa following the maternal-to-zygotic transition (Coşacak et al., 2018). A side-by-side analysis of small RNAs in total RNA and fractionated RNAs showed the dynamic re-distribution of miRNA in the cytoplasm irrespective of a potential change in the transcriptional output. Molotski and Soen (2012) observed a similar differential polysomal occupancy of miRNAs between human embryonic stem cells and foreskin fibroblast cells (Molotski and Soen, 2012). Even more interestingly, differential polysomal association of miRNAs was reported to involve the formation of diverse miRNA effector complexes regulated by extracellular signals (Wu et al., 2013). One interpretation of this finding is that the stimulus-mediated switch from a polysomal to a non-polysomal miRISC state may result in the formation of different effectors. Such a scenario is possible, for example, when different complexes are formed during de-repression from miRNA-mediated down-regulation (Orang et al., 2014). Then, the remaining question is, considering the polysomal and non-polysomal sites, which one is the actual site of action and which one the site of relief? One caveat of small RNA profiling approaches is that not all miRNAs or AGO proteins may be bound to each other, suggesting that miRNAs may be bound to their targets in the absence of AGO proteins (Wang et al., 2009; Janas et al., 2012). One should be careful in the intepretation of miRNA location when using RNA-seq data to localize functionally active miRISC after sub-cellular fractionation. Thus, the quantitation of all existing miRNAs could not necessarily represent the functional miRISC complexes. AGO-bound miRNAs should be analyzed in any cellular sub-compartments to isolate potentially functional miRISC complexes, avoiding merely mRNA-bound, AGO-free miRNAs.

An interesting question regarding miRISC localization is how various miRISCs are assembled and cargoed in a selective manner to polysomal or non-polysomal sites. There are several miRISC accessory proteins that may modulate the intracytoplasmic fate of miRISCs (Krol et al., 2010). For example, HSP90 is a crucial regulator of AGO2 localization in non-polysomal foci such as PBs and SGs (Pare et al., 2009). Also, AGO2 post-translational modifications, such as hydroxylation or phosphorylation, may modulate the localization of miRISCs in PBs (Qi et al., 2008; Zeng et al., 2008). It is unknown at this point whether specific intracytoplasmic localization of miRISCs is associated with unequal target abundance in the cytoplasm. Although Coşacak et al. (2018) did not find any correlation between target abundance and miRNA re-arrangement, Molotski and Soen (2012) reported the significance of the seed sequence in the polysome occupancy (Molotski and Soen, 2012; Coşacak et al., 2018). Along the same line, target-dependent biogenesis of miR-122 during stress reversal has been reported in HEK293 cells (Bose and Bhattacharyya, 2016). Thus, it needs to be resolved whether it is the mRNA target location that determines the miRISC location or vice versa. Assuming that the switch between polysomal and non-polysomal fractions under two different phenotypes is because of selective trafficking of target mRNAs (not because of the difference in mRNA identity), an intracellular or extracellular stimulus should be responsible for re-localization of miRISCs. Such a scenario could be facilitated through post-translational modification of protein components or post-transcriptional editing of RNA component of RISC. Indeed, AGO2 phosphorylation at S387 results in its re-localization to PB, favoring translational repression (Horman et al., 2013).

## Concluding Remarks

miRISC complexes are distributed throughout the cell, both in the nucleus and cytoplasm depending on the cell type and cellular phenotype. However, more evidence exists for the involvement of PBs in AGO2-mediated regulation of miRISC activity. Interestingly, the cytoplasmic location of miRISCs appears to correlate with their fate. For example, ER harbors miRISCs, which appear to regulate the translation of mRNAs destined for ER and golgi while P bodies house miRISCs associated with mRNAs targeted for degradation. An interesting observation from recent studies is the dynamic changes in the localization of miRISCs between polysomal and non-polysomal (e.g., SGs or P bodies) structures. Although small RNA profiling following cytoplasmic fractionation provides some indirect evidence for such re-arrangement of miRISC complexes, more direct evidence would require the tracing of labeled miRNAs under two different states (e.g., two development states or health states) to eliminate the target heterogeneity as the potential cause of re-localization. More importantly, it begs for more research to delineate the protein and/or miRNA components of miRISCs that are critical for shuffling miRISCs between polysomal and non-polysomal complexes as part of miRISC regulation.

## Author Contributions

BA conceived, designed, and wrote the paper. İE participated in designing, revising, and coordination. All authors reviewed and approved the final version.

## Conflict of Interest Statement

The authors declare that the research was conducted in the absence of any commercial or financial relationships that could be construed as a potential conflict of interest.
